# Midazolam versus midazolam-promethazine combination for oral sedation in third molar surgery: A randomized split-mouth trial

**DOI:** 10.4317/medoral.28014

**Published:** 2026-04-19

**Authors:** Danilo-de-Moraes Castanha, Thayane Celina Silva Lessa, Emerllyn Shayane Martins-de-Araújo, Taysnara Ismaely-de-Andrade, Gabrieli Maria Barbosa Beserra, Eduarda Gomes Onofre-de-Araújo, Fábio Andrey-da-Costa Araújo, Emanuel Savio-de-Souza Andrade

**Affiliations:** 1 University of Pernambuco, Campus Santo Amaro (Faculty of Dentistry of Pernambuco), Graduate Program in Dentistry, Recife, Pernambuco, Brazil; 2 Unifavip Wyden, University Center of Paraíba, João Pessoa, Paraíba, Brazil; 3 Federal University of Paraíba, Graduate Program in Dentistry, João Pessoa, Paraíba, Brazil

## Abstract

**Background:**

Oral sedation is widely utilized in dental surgical practice for anxiety management during third molar extractions. Midazolam is one of the most frequently employed drugs; however, the clinical benefits of its association with promethazine have not yet been clearly established.

**Material and Methods:**

This is a randomized, double-blind, split-mouth clinical trial. Eighteen anxious patients were included, all with indications for bilateral extraction of impacted or semi-impacted mandibular third molars, presenting similar surgical difficulty on both sides. In one session, patients received 15mg of midazolam alone (Group A), and in another, 15mg of midazolam combined with 25mg of promethazine (Group B). Sedation levels (Ramsay scale), anxiety (Corah scale), and physiological parameters were evaluated.

**Results:**

There was no statistically significant difference in the depth of sedation between the groups (p&gt;0.05). Oxygen saturation and heart rate showed slight, statistically significant variations at specific time points in Group B (p&lt;0.05), although without relevant clinical repercussions. Blood pressure and respiratory rate remained stable across both protocols.

**Conclusions:**

The association of promethazine with midazolam did not increase sedative efficacy compared to midazolam alone. However, it demonstrated clinical safety, proving to be a viable alternative in sedative drug protocols.

## Introduction

Dental anxiety represents a challenge in clinical practice, directly interfering with treatment adherence and patient comfort, especially in surgical procedures such as third molar extractions. This condition is frequently associated with fear of pain, previous negative experiences, and phobias related to instruments such as needles and drills, which can trigger adverse physiological reactions, including anxiety attacks, hyperventilation, increased blood pressure, and the aggravation of systemic comorbidities (3 , 4).

In this scenario, oral conscious sedation stands out as an accessible and viable approach in an outpatient setting. Midazolam, a widely used benzodiazepine, is considered the drug of choice for promoting moderate sedation in dental procedures, acting through the enhancement of the inhibitory effect of GABA in the central nervous system, which results in relaxation, drowsiness, and anxiety reduction (5). Despite its efficacy, the drug can be associated with adverse effects, such as paradoxical reactions, respiratory changes, and cardiac arrhythmias (6 , 7).

The combination of drugs with sedative potential has been extensively investigated in oral surgery, especially in third molar extractions, with the objective of achieving a synergistic effect and greater patient comfort. In this context, promethazine, a low-cost and easily accessible antihistamine, exhibits sedative, hypnotic, antisialogue, and antiemetic properties. In dental practice, its use has been described both as a monotherapy and in association with other drugs (8).

Despite promising results in pediatric populations, there is still a scarcity of scientific evidence regarding the efficacy of the oral combination of midazolam and promethazine in adults undergoing third molar extractions. Thus, this study aimed to compare the efficacy of the promethazine-midazolam association with the isolated use of midazolam for the sedation of patients undergoing mandibular third molar extractions.

## Material and Methods

Study Design

This is a randomized, double-blind, controlled, split-mouth clinical trial conducted at a higher education institution in the region of Pernambuco, Brazil, involving patients treated at the Oral and Maxillofacial Surgery and Traumatology outpatient clinic with indications for bilateral mandibular third molar extractions. The depth of sedation was evaluated using the Ramsay Scale, considering different drug protocols. Additionally, hemodynamic parameters, including heart rate, systolic and diastolic blood pressure, respiratory rate, and oxygen saturation, were monitored during the preoperative, intraoperative, and postoperative periods to compare the protocols. The study was conducted and reported in accordance with the CONSORT checklist and its extension for randomized clinical trials (9), having received approval from the Research Ethics Committee CEP HUOC/PROCAPE (CAAE: 79552823.0.0000.5207).

Study Sample and Group Randomization

The sample consisted of 18 volunteer patients recruited from the School Clinic of the Faculty of Dentistry of Pernambuco and the Oral and Maxillofacial Surgery and Traumatology outpatient clinic of the institution. All participants had indications for the extraction of impacted or semi-impacted mandibular third molars, in homologous positions and with an equivalent degree of surgical difficulty, according to the Pernambuco Index (10). Individuals aged 18 to 50 years who presented fear or anxiety related to the surgical procedure, defined by a score of 11 on the Corah Scale, and classified as healthy patients or those with controlled systemic disease, were included. Patients with contraindications to the use of benzodiazepines; a history of severe respiratory diseases, obstructive sleep apnea, congestive heart failure, hepatic or renal failure; previous use of central nervous system depressant drugs or medications with potential interaction with the evaluated drugs; pregnant or lactating women; individuals with a history of drug dependence or alcoholism; hypersensitivity to any component of the medications, substances, or materials used; as well as patients with severe or uncompensated systemic diseases, blood dyscrasias, or chronic pain, were excluded.

Sample size calculation was based on Student's t-test for paired samples, considering a significance level of 5% and a statistical power of 80%, resulting in a minimum sample of 14 participants. An additional 25% was included to compensate for potential dropouts, totaling 18 patients in the study. Each participant underwent two surgical procedures, one on each side, with the order of interventions previously randomized using Random.org software. Group allocation was performed by drawing from opaque envelopes: Group A received 15mg of midazolam combined with a placebo, while Group B received 15mg of midazolam combined with 25mg of promethazine. In the second procedure, both the operated side and the drug protocol were reversed (split-mouth). The study was conducted in a double-blind manner, with medications packaged indistinguishably to ensure the blinding of both the surgeon performing the procedure and the outcome evaluator.

Clinical Procedures and Data Collection

All procedures were performed by an experienced surgeon at the Oral and Maxillofacial Surgery and Traumatology Research Center of the Faculty of Dentistry of Pernambuco. Patients maintained a light fast for up to 2 hours before surgery and received, one hour prior to the procedure, 8mg of dexamethasone, 1g of amoxicillin, 15mg of midazolam, and the medication corresponding to the randomization (25mg of promethazine or a placebo). Local anesthesia was performed using 2% lidocaine with 1:100,000 epinephrine for inferior alveolar, lingual, and buccal nerve blocks. Surgical access included an intrasulcular incision associated with a posterior releasing incision, with osteotomy performed using tapered fissure burs under saline irrigation. Following extraction, suturing was performed with 4-0 nylon thread. In the postoperative period, patients received instructions regarding diet and oral hygiene, the use of 0.12% chlorhexidine mouthwashes, and analgesia with 600mg of ibuprofen (every 8 hours) and 1g of dipyrone (every 6 hours) for 3 days.

Data collection was performed by an independent researcher after ethical approval and the attainment of informed consent. Anxiety levels (Corah Scale), sedation (Ramsay Scale), and hemodynamic parameters (heart rate, blood pressure, and oxygen saturation) were evaluated and recorded during the preoperative, intraoperative, and postoperative periods. Demographic data (age, gender, and body weight) were also collected. The average time for protocol execution was approximately 45 minutes per patient.

Data Analysis

Data were analyzed using SPSS software (version 20.0) through descriptive and inferential statistics. The normality of the variables was assessed using the Shapiro-Wilk test. Variables with a normal distribution were analyzed using Student's t-test for paired samples, while those without a normal distribution were evaluated using the Wilcoxon signed-rank test. Intra-individual comparisons between the sedation protocols (isolated midazolam and midazolam combined with promethazine) included sedation levels (Ramsay Scale), anxiety (Corah Scale), and hemodynamic parameters at different stages of the procedure. Categorical variables were analyzed using Chi-square or Fisher's exact tests, as appropriate. The significance level was set at 5% (p&lt;0.05), with a minimum statistical power of 80%.

To reduce the risk of type I error due to multiple paired comparisons across time points, p-values were adjusted using the Holm-Bonferroni method within each outcome variable (11). In addition to statistical significance, effect sizes were calculated (Cohen's dz for paired t-tests and r for Wilcoxon signed-rank tests), along with 95% confidence intervals, to enhance the clinical interpretability of the findings.

## Results

Participants were recruited and underwent surgical procedures between November 2024 and February 2025. The sample consisted of 18 patients, predominantly female, with a mean age of 24.5±4.41 years. The Ramsay Scale was used to measure the sedation achieved with the proposed protocols (Table 1).

Table 1The group that received midazolam alone (Group A) presented a median of 2.00 (min-max: 2.00-3.00), with a mean of 2.44 (SD=0.62), while the group that received the combination of midazolam and promethazine (Group B) presented a median of 2.00 (min-max: 2.00-3.00), with a mean of 2.33 (SD=0.49). The paired comparison between groups, performed using the Wilcoxon test, showed no statistically significant difference (p=0.586).

Regarding peripheral oxygen saturation (SpO2), although the medians remained at 99% in both groups, a statistically significant difference was observed in the mean values five minutes after the end of surgery, with a mean of 98.94% in Group A and 98.67% in Group B. The paired comparison between the protocols, performed using the Wilcoxon test, demonstrated a statistically significant difference (p=0.025). However, the values remained within normal physiological limits at all evaluated time points, without relevant clinical repercussions (Table 2).

**Figure 1:**
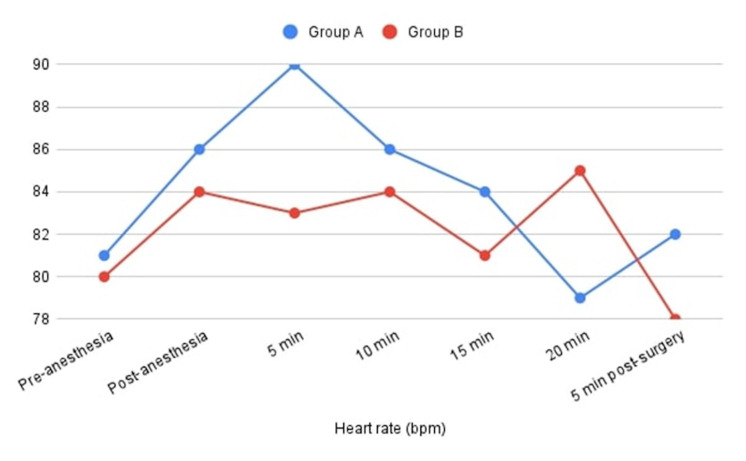
Evolution of heart rate over time.

Table 2Analysis of systolic and diastolic blood pressure, evaluated during the preoperative, post-anesthesia, intraoperative, and postoperative periods, showed no statistically significant differences between the groups. At five minutes into the procedure, the systolic blood pressure presented a mean of 117.9mmHg in Group A and 114.2mmHg in Group B (p=0.185), while the preoperative diastolic blood pressure was 77.4mmHg and 75.3mmHg, respectively (p=0.477) (Table 2). Heart rate showed a statistically significant difference at a single time point, five minutes after the start of surgery. Group A presented a mean of 90.2 bpm and Group B a mean of 83.0 bpm (Table 2). A significantly lower heart rate was observed in Group B at 5 minutes compared to Group A (t(17)=3.06; adjusted p=0.049), with a mean difference of 7.22 bpm (95% CI: 2.24-12.20). The effect size was moderate to large (d=0.72), suggesting potential clinical relevance of the observed reduction, despite the borderline statistical significance. At all other evaluated moments, heart rate values showed no statistically significant differences between the groups (Figure 1).

1

Regarding respiratory rate, no relevant statistical differences were observed between the groups at any of the evaluated time points. The mean values remained within normal physiological parameters, remaining similar between the protocols throughout the entire procedure (Table 2).

## Discussion

A randomized split-mouth clinical trial was conducted to determine if the addition of promethazine to oral midazolam would enhance sedation depth or improve physiological stability in adults undergoing extraction of mandibular third molars. The findings did not demonstrate superior sedative efficacy with the combination protocol, while both regimens maintained physiological stability within normal limits. These results suggest that, under the conditions evaluated, the addition of promethazine did not provide measurable clinical advantage over midazolam alone.

Regarding the depth of sedation, measured by the Ramsay Scale, no statistically significant difference was observed between the groups, although the group that received midazolam alone presented a slightly higher mean. This finding contradicts the hypothesis that the combination of promethazine would potentiate the sedative effect through synergistic action, as midazolam alone may already achieve a pharmacodynamic plateau sufficient for moderate oral sedation in healthy adults. A possible explanation may lie in the pharmacological profile of the medications used. Midazolam has a rapid onset of action and a well-established sedative effect when administered orally (12). On the other hand, promethazine, despite its sedative properties, may present variable oral absorption and a milder effect in adults (13 , 14), which may explain the absence of measurable additive benefit in this clinical context.

Barzegari et al. (15) had similar findings. They assessed pediatric patients undergoing computed tomography and found no significant difference in sedation depth between midazolam alone and midazolam plus promethazine. A faster induction time was noted with the combined protocol, but sedation levels were equivalent. This information should be used with caution since pediatric data may not apply to adults due to differences in drug metabolism, central nervous system sensitivity, and body composition with age that can affect both pharmacokinetics and pharmacodynamics. Pediatric evidence indicates possible adjuvant effects under certain conditions, yet such benefits do not necessarily result in clinically relevant improvements for adult oral surgery patients.

On the other hand, a clinical trial comparing two oral sedation protocols in children undergoing dental procedures, chloral hydrate/midazolam and promethazine/midazolam, identified no significant differences in sedation levels between the groups (8), indicating that both regimens were effective in achieving the desired sedation. However, the authors observed a lower occurrence of postoperative problems in the group that received the promethazine/midazolam combination, suggesting a possible advantage in terms of recovery and post-procedure comfort. These findings indicate that, although both combinations are effective, the promethazine/midazolam association may present a more favorable profile of postoperative adverse effects, making it a viable and safe alternative, especially for short-duration outpatient procedures.

Unlike the previously presented studies, promethazine was evaluated in combination with haloperidol and compared to the efficacy of midazolam alone (16). The authors observed that midazolam promoted significantly faster sedation, although both protocols were effective in controlling agitation. This result can be explained by the pharmacokinetic profile of midazolam, which, being highly liposoluble, presents a rapid onset of action and a relatively short half-life (12). In contrast, the haloperidol-promethazine combination, although effective, demonstrated a slower onset of action and a higher occurrence of mild extrapyramidal effects. These findings reinforce that while promethazine can act as an adjuvant in sedation, midazolam proves more advantageous regarding induction time, a relevant aspect also in dental procedures that require fast and safe sedation. Similar to what was observed in the present study, the combination of midazolam with promethazine maintained hemodynamic stability and clinical safety, but did not result in deeper sedation or faster onset of action compared to the use of midazolam alone. It is further discussed that promethazine can exert a sedative effect even when used alone, as demonstrated in a comparative study between promethazine and midazolam as anesthetic premedication in children aged 3 to 9 years undergoing surgical procedures (17). The authors observed that both drugs were effective in reducing anxiety, with high levels of acceptance and perceived efficacy; however, midazolam presented a faster and more predictable onset of action, favoring the child's separation from parents and the acceptance of anesthetic induction. Regarding safety, the aforementioned study demonstrated stability in blood pressure and respiratory rate throughout the evaluation period, a finding that corroborates the results of the present study, in which no statistically significant differences were observed in systolic and diastolic blood pressure values, nor in respiratory rate between the evaluated protocols. Although a statistically significant difference was identified in peripheral oxygen saturation, this variation was slight, without clinical relevance and within normal physiological parameters. Thus, the results reinforce the safety profile of the combination of midazolam and promethazine, even in the face of small statistical variations in the monitored parameters.

A statistically significant reduction in heart rate was found at five minutes after the beginning of surgery in the promethazine-containing group. Even though the adjusted p-value was borderline, the size of difference (mean reduction of 7.22 bpm; 95% CI: 2.24-12.20) and moderate-to-large effect size can indicate that this finding has some clinical relevance. From a physiological viewpoint, a lower heart rate during early surgical phases may indicate increased anxiolytic activity or autonomic modulation. However, this finding was not sustained at later time points, and all values remained within normal physiological limits. Thus, while the reduction in heart rate may indicate a modest autonomic effect of promethazine when co-administered with midazolam, its clinical significance in healthy adults appears to be limited.

It is important to highlight that, although the current findings of the present study reveal clinical safety and hemodynamic stability in the parameters observed with both protocols, the impact of the drug combination on sedation levels can be considered insufficient. This observation reinforces that, although drug combinations for sedative purposes may be useful in specific cases, rational and individualized use remains the best approach (18).

Thus, the data obtained contribute to expanding the understanding of the use of promethazine for sedation in adults, a field that remains under-explored in the literature. However, although an a priori sample size calculation was performed, the relatively small sample may have limited the statistical power to detect subtle or clinically small differences between protocols, particularly across multiple perioperative time points and outcomes. This limitation highlights the need to expand the sample to obtain more precise and generalizable results for the population. Additionally, the absence of subjective outcome measures (e.g., patient and surgeon satisfaction), the non-evaluation of postoperative recovery time, and the potential influence of memory or learning effects inherent to the split-mouth design may have limited a broader clinical interpretation of the findings.

## Conclusions

The combination of promethazine and midazolam for oral sedation in third molar extractions did not result in a significant increase in sedation compared to the use of midazolam alone. Despite small variations in hemodynamic parameters, the combination proved to be clinically safe.

## Figures and Tables

**Table 1:** Table Description of the study variables.

Variable	Data	%
Gendera		
Male	4	22.2
Female	14	77.8
Age, meanb	24.5±4.41	-
Ramsay Scalec		
Group A	2 (2-3)	-
Group B	2 (2-3)	-
Corahc	11 (11-17)	-

a, n (%): Absolute frequency and relative frequency. b: Mean±standard deviation. c: Median (minimum-maximum)

**Table 2:** Table Paired comparisons of physiological parameters between sedation protocols over time.

Variable	n	Group A	Group B	Paired difference	Effect size	95% CI	Adjusted p-value
Oxygen saturation (SpO2,, %)a							
Pre-anesthesia	18	99 (98-99)	99 (97-99)	Δ=0.44	0.63	0.11 to 1.13	0.096
Post-anesthesia	18	99 (97-99)	99 (98-99)	Δ=0.00	0.00	-0.46 to 0.46	1.000
5 minutes	18	99 (96-99)	99 (97-99)	Δ=-0.05	-0.05	-0.51 to 0.41	1.000
10 minutes	18	99 (97-99)	99 (98-99)	Δ=-0.14	-0.18	-0.71 to 0.34	1.000
15 minutes	18	99 (96-99)	99 (98-99)	Δ=-0.42	-0.37	-1.13 to 0.40	1.000
5 minutes post-surgery	18	99 (98-99)	99 (98-99)	Δ=0.27	0.60	0.09 to 1.09	0.100
Systolic blood pressure (mmHg)b							
Pre-anesthesia	18	121±11.11	121±9.5	Δ=0.16	0.02	-0.43 to 0.48	0.921
Post-anesthesia	18	117±13.9	116±12.7	Δ=0.83	0.08	-0.38 to 0.54	1.000
5 minutes	18	118±15.82	114±9.77	Δ=3.66	0.32	-0.15 to 0.79	1.000
10 minutes	14	119±10.59	116±9.72	Δ=2.28	0.29	-0.24 to 0.82	1.000
15 minutes	6	118±6.83	118±6.12	Δ=-1.88	-0.27	-0.93 to 0.39	1.000
20 minutes	2	118±4.24	117±2.12	Δ=1.50	0.70	-1.00 to 2.23	1.000
5 minutes post-surgery	18	118±12.2	116±11.9	Δ=1.72	0.18	-0.28 to 0.64	1.000
Diastolic blood pressure (mmHg)c							
Pre-anesthesia	18	77.4±10.9	75.3±14.7	Δ=2.17	0.17	-0.29 to 0.63	1.000
Post-anesthesia	18	77.1±14.3	72.4±9.3	Δ=4.67	0.37	-0.10 to 0.85	0.762
5 minutes	18	74.2±11.26	71.8±8.69	Δ=2.44	0.28	-0.19 to 0.75	1.000
10 minutes	14	75.1±8.12	74.1±6.95	Δ=1.00	0.13	-0.39 to 0.66	1.000
15 minutes	6	75.5 (73.5-78.5)	74.3 (73.5-78.5)	Δ=1.17	0.16	-0.64 to 0.96	1.000
5 minutes post-surgery	18	71.5±15.91	72.6±8.63	Δ=-1.06	-0.05	-0.52 to 0.40	1.000
Heart rate (bpm)d							
Pre-anesthesia	18	81.4±8.5	80.2±10.4	Δ=1.22	0.10	-0.35 to 0.56	1.000
Post-anesthesia	18	86.4±14	84.3±13.7	Δ=2.11	0.16	-0.30 to 0.62	1.000
5 minutes	18	90.3±11.8	83.1±11.8	Δ=7.22	0.72	0.19 to 1.23	0.049
10 minutes	13	86.2±9.5	84.2±11.9	Δ=1.92	0.18	-0.36 to 0.72	1.000
15 minutes	6	84.7±8.04	81.8±7.14	Δ=2.83	0.31	-0.52 to 1.12	1.000
20 minutes	2	79±1.41	81.5±3.54	Δ=-2.50	-1.17	-3.08 to 0.85	1.000
5 minutes post-surgery	18	82.4±9.39	78.4±11	Δ=4.06	0.39	-0.09 to 0.87	0.672
Respiratory rate (brpm)c							
Pre-anesthesia	18	16 (12-22)	15.5 (12-26)	Δ=0.00	0.00	-0.46 to 0.46	1.000
Post-anesthesia	18	20.1±3.4	19.9±2.6	Δ=0.16	0.06	-0.39 to 0.52	1.000
5 minutes	18	19.7±3.77	19.1±3.83	Δ=0.55	0.16	-0.30 to 0.62	1.000
10 minutes	14	18.8±3.31	18.7±2.43	Δ=0.07	0.02	-0.49 to 0.54	1.000
15 minutes	6	17.2±3.43	17.3±1.86	Δ=-0.16	-0.04	-0.84 to 0.75	1.000
5 minutes post-surgery	18	18.5±3.09	17.3±2.59	Δ=1.22	0.32	-0.15 to 0.79	1.000

2

## Data Availability

Declared none.
